# Activation of PI3K/p110α in the Lung Mesenchyme Affects Branching Morphogenesis and Club Cell Differentiation

**DOI:** 10.3389/fcell.2022.880206

**Published:** 2022-05-23

**Authors:** Haiting Dai, Mingli Zhu, Wenya Li, Guohui Si, Yiming Xing

**Affiliations:** State Key Laboratory of Agrobiotechnology, College of Biological Science, China Agricultural University, Beijing, China

**Keywords:** lung, PI3K, p110α, mesenchymal cell, branching morphogenesis, club cell differentiation, *Isl1-Fgf10-Sox9*, *Pten-Hes1*

## Abstract

Epithelial–mesenchymal interaction is required for normal growth, morphogenetic patterning, and cellular differentiation in developing lungs. Various signaling pathways have been defined in establishing the patterning of this branched organ. The phosphoinositide-3-kinase (PI3K) signaling plays an important role in disease pathogenesis but remains largely uncharacterized in embryonic development. In this study, we activated a specific catalytic subunit of PI3K catalytic enzymes, Class IA p110α (p110α), in the embryonic lung mesenchyme using the *Dermo1-Cre* mouse. Activation of p110α promoted branching morphogenesis and blocked club cell differentiation in both proximal and distal airways. Mechanistically, the LIM homeodomain gene Islet-1 (*Isl1*), fibroblast growth factor 10 (*Fgf10*), and SRY (sex-determining region Y)-box9 (*Sox9*) were found to be downstream targets of p110α. The significantly increased expressions of *Isl1*, *Fgf10,* and *Sox9* resulted in the stimulation of branching in mutant lungs. Activation of p110α-mediated signaling also increased the expression of phosphatase and tensin homolog deleted on chromosome 10 (*Pten*) and hairy/enhancer of split 1 (*Hes1*), which in turn blocked club cell differentiation. Thus, the signaling pathway by which PI3K/p110α-regulated epithelial–mesenchymal interactions may entail *Isl1*–*Fgf10*–*Sox9* and *Pten*–*Hes1* networks, which consequently regulate branching morphogenesis and club cell differentiation, respectively.

## Introduction

During embryogenesis, the mammalian lung is derived by branching morphogenesis to form an effective gas exchange organ. Traditionally, the development of the bronchopulmonary tissue has been defined in five histological stages: embryonic, pseudoglandular, canalicular, saccular, and alveolar stages (Metzger et al., 2008; Mullassery and Smith, 2015). In the mouse, the specification of the lungs starts around embryonic day (E) 9.0. By E9.5, evagination of the epithelium forms the trachea and two lung buds (Cardoso, 2001; Morrisey and Hogan, 2010). The trachea separates from the esophagus in the embryonic stage (E9.5–E12.5) (Boggaram, 2009; Morrisey and Hogan, 2010). In E12.5–E16.5, to establish a tree-like airway network, the lung buds conduct a strictly regulated branching process. In E16.5–postnatal day 5 (canalicular and saccular stages), the branch terminuses develop alveolus to form a large surface area in preparation for air exchange to the blood at birth. In P0–P14 (alveolarization stage), the alveolus is fully matured (Frank et al., 2016). Through all stages, signaling pathways act in concert with one another between the endodermal epithelium and mesenchyme, contributing to normal growth, morphogenetic patterning, and cellular differentiation in the developing lung (Kolobaric et al., 2021).

After the early budding of the foregut endoderm to form the main bronchi, the airway tips rapidly grow into the surrounding mesenchyme to establish the tree-like branching structure. Branching morphogenesis is mediated by changes in cell behaviors, such as cell size, shape, division, and motility (Zhu and Nelson, 2012). These cellular effects are highly regulated by various signaling pathways, including the FGF (fibroblast growth factor), Wnt, Shh (sonic hedgehog), and BMP (bone morphogenetic protein) pathways (Cardoso and Whitsett, 2008). Although the mechanisms involved in forming new branch points are still not identified, the present literature has documented that the signaling networks in epithelial and mesenchymal cells are required by both branching morphogenesis and cell differentiation. FGF10, a member of the FGF superfamily, originates from the distal lung mesenchyme and is regulated by the SHH and BMP4 pathways (Ohuchi et al., 2000). Disruption of FGF10 in mice blocked lung development below the trachea (Kato and Sekine, 1999). In contrast to FGF10, SHH is present in the lung epithelium, with a most profound expression in the distal tips. Deletion of SHH in mice also showed an abnormal patterned lung (Litingtung et al., 1998) and extension of FGF10 expression (Pepicelli et al., 1998). Similar to SHH, BMP4 is also highly expressed in the epithelial cells around the distal tips. Overexpression of BMP4 results in lung hypoplasia and distension of terminal airspaces (Bellusci et al., 1996). The FGF10–Shh and FGF10–BMP4 interactions suggest that a complex signaling network in the epithelium and mesenchyme controls branch formation and outgrowth.

During branching, the lung endoderm starts to make cell fate decisions and differentiates into specialized cell types along a proximal–distal axis. The Sry-related HMG box proteins, SOX2 and SOX9, define the proximal–distal patterning. SOX2 is located in the proximal epithelium, whereas SOX9 is strictly expressed in the distal epithelium (Gontan et al., 2008; Rockich et al., 2013). The specific distribution of Sox2 and Sox9 leads to proper cell proliferation and differentiation (Okubo et al., 2005). When the SOX2^+^ progenitors give rise to airway secretory cells, neuroendocrine cells, mucosal cells, and ciliated cells, the SOX9^+^ progenitors differentiate into alveolar type I and type II cells (Tompkins et al., 2011; Herriges and Morrisey, 2014). Although multiple studies have shown that signaling from the mesenchyme plays a critical role in the airway epithelial cell differentiation, neither the heterogeneity of the SOX2^+^ and SOX9^+^ progenitor populations nor the molecular mechanisms underlying their formation and differentiation are fully identified.

PI3Ks (phosphatidylinositol 3-kinases) are a family of lipid kinases and can be divided into three subclasses based on their specificity in structure and regulations (Noorolyai et al., 2019). The important role of the PI3K signaling network has been defined in many physiological processes, including cell growth, proliferation, differentiation, motility, and survival (Martini et al., 2014). Among the three subclasses, Class I PI3Ks contain a p110 catalytic subunit (p110α, *β*, or p110γ), and a p85 regulatory subunit (Zhao and Vogt, 2008). In the past decades, substantial advances in understanding the importance of PI3K in human cancer have been obtained by analyzing Class I PI3Ks and, specifically, the p110α isoform (Fruman et al., 2017). It is now appreciated that PI3K is also a major player in controlling normal organogenesis including branching morphogenesis in the mammary gland, kidneys, salivary gland, and prostate (Zhu and Nelson, 2012). Evidence from mouse embryonic lung culture experiments has suggested that the disruption of PI3K decreased the number of buds, the diameter of the developing airways, and epithelial cell proliferation (Srinivasan et al., 2009). Although the PI3K pathway is important for lung development, various roles are still needed to be systematically examined.

In this study, we investigated the role of PI3K in embryonic lung development by generating mice that constitutively express an active p110α (p110*) in mesenchymal cells via a *Dermo1-Cre* driver mouse line. The results show that PI3K signaling via p110α regulates branching morphogenesis and club cell differentiation. *Isl1*, *Fgf10,* and *Sox9* expressions were stimulated in mutant lungs, which in turn were associated with increased branches. The expansion of airway epithelial progenitors in the mutant lung led to an impeded club cell differentiation in both proximal and distal airways through *Pten*-regulated *Hes1* expression. To the best of our knowledge, our work provides the first comprehensive evidence that impacts PI3K signaling via p110α on epithelial–mesenchymal interactions that are required for embryonic lung development.

## Materials and Methods

### Experimental Animals

P110* mice were generated as previously described (Srinivasan et al., 2009) and purchased from Jackson Laboratory (strain name: C57BL/6-*Gt(ROSA)26Sor* ^tm7(Pik3ca*,EGFP)Rsky/J^, stock number: 012343, also known as R26Stop^FL^P110*). In these mice, the R26Stop^FL^P110* conditional allele is targeted to the *Gt(ROSA) 26Sor* locus and has a loxP-flanked Neo-STOP cassette preventing transcription of P110α and EGFP. These mice allow an inducible expression of activated PIK3 heterodimer activity. *Dermo1-Cre* and *ROSA* ^*mTmG*^ mice were gifts from Dr. Parviz Minoo (University of Southern California, United States). P110*/* mice were crossed with *Dermo1-Cre* mice to obtain P110*/+; Dermo1-are mice. All animals were housed up to the standard protocol approved by the Beijing Association on Laboratory Animal Care (Beijing, China), and all animal studies were conducted according to the protocol (AW81801202-3-2) from China Agriculture University.

### Embryonic Lung Isolation

Timed-pregnant mice were sacrificed on E12.5, E15.5, and E17.5. The embryos were released from the uterus and lungs and removed under a 3D dissecting microscope (DUMONT, 0208-5-PO) and fixed with 4% PFA for 4 h for histological analyses or collected in RNase-free centrifuge tubes for RNA and protein analyses.

### Histology and Immunohistochemistry

For the preparation of paraffin sections, all dissected lung tissues were fixed in 4% PFA. Hematoxylin–eosin (H&E) staining was performed according to the standard procedures for morphological examination. Immunofluorescence (IF) staining was performed as previously described (Sridurongrit et al., 2008). IHC kits were purchased from ZSGB-BIO, China (PV-9001). Primary antibodies used in the experiments are listed in Supplemental Table S1.

### RNA Extraction and Quantitative Real-Time PCR

Total RNA from lung tissues was extracted with the TRIzol reagent (Invitrogen, Life Technologies, China). Reverse transcription was conducted according to the manufacturer’s instructions (Vazyme, R333-01, China). Real-time PCR analysis was performed by using 2xM5 HiPerSYBR Premix EsTaq with Tli RNaseH (Mei5 Biotechnology, China) and a Light Cycler 480 real-time PCR system (Roche). The expression of *β-actin* was used to normalize target gene expression. The sequence of primers is listed in Supplementary Table S2.

### Western Blot

Lung tissues were collected and frozen in liquid nitrogen. Total proteins were extracted by using a RIPA reagent kit (Beyotime, China) containing 1 mM PMSF (Beyotime, China) and a PhosSTOP EASY kit (Roche, Switzerland). An equal amount of proteins from each sample was loaded and separated using SDS/PAGE gels, then transferred to Immobilon-P transfer membranes, and stained with primary antibodies. Antibodies against the target proteins are listed in Supplemental Table S1.

### Cell Apoptosis

The apoptotic cells were detected using a TUNEL Apoptosis Assay Kit (Beyotime Biotechnology Co., Shanghai, China). Images were captured with a fluorescence microscope.

### Cell Counting

After immunostaining, images were taken by using an Olympus BX53 microscope. The cells were quantified with ImageJ software or Adobe Photoshop (CC 2019). More than 10 random areas per section were counted under the ×20 objective.

### Mesenchymal Cell Isolation

Lungs from mutant or control mice were dissected at E12.5 and treated with Dispase (50 U/ml, Beyotime Biotechnology Co., Shanghai, China) at 4°C for 20 min. Mesenchymal cells around the distal lung tips were removed with tungsten needles and collected for protein extraction.

### Statistical Analysis

Data are presented as mean ± SD as indicated. *p*-values were calculated using SPSS 16.0 software. A *p* value <0.05 was considered significant, whereas >0.05 was assigned as NS. Statistical significance between the two groups was carried out by using an unpaired two-tailed Student’s *t*-test.

## Results

### Mesenchymal-Specific Activation of p110α in Murine Lungs

**FIGURE 1:**
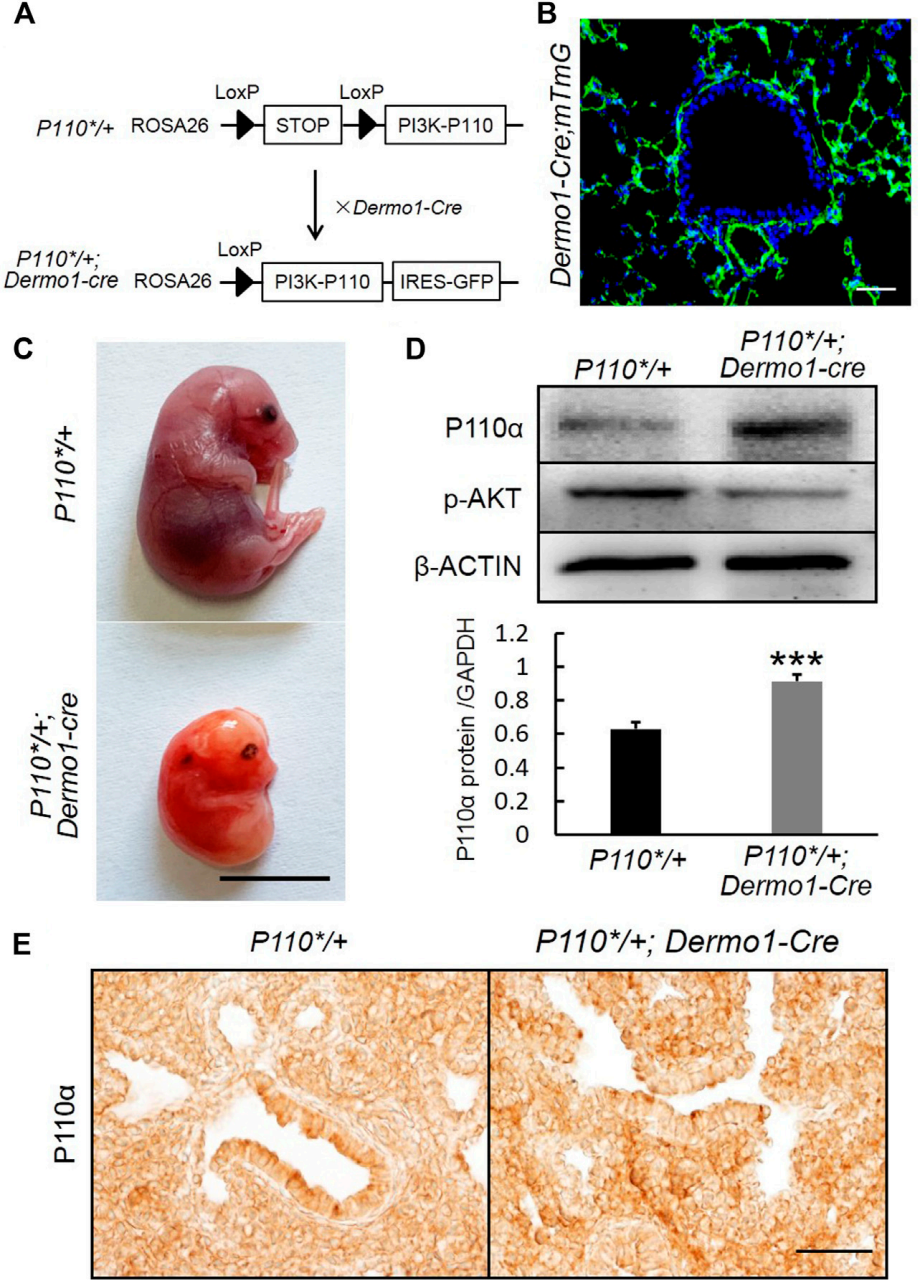
Generation of P110*/+; Dermo1-cre mice. **(A)**. P110*/+; *Dermo1-cre* was generated by crossing P110*/* with *Dermo1-Cre* mice. **(B)**. An IF staining analysis showed GFP-labeled mesenchymal cells in *Dermo1-Cre; ROSA* ^*mTmG*^ mice. Scale bar: 50 μm. **(C)**. Gross morphology of E20.5 control and P110*/+; *Dermo1-cre* embryos. Scale bar: 1 cm. **(D)**. The expression of p110α and phosphorylated AKT at E12.5 were analyzed by using western blot. The western blot result for p110α was quantified with ImageJ software. **(E)**. Immunolocalization of P110α in E17.5 control and P110*/+; Dermo1-cre lungs. Scale bar: 50 μm.

P110α, the catalytic subunit of PI3K, plays a key role in cell survival, growth, and proliferation, as well as differentiation, regeneration, hypertension, and development of cancer (Klippel et al., 1996). In order to study the regulation of mesenchymal PI3K signaling during embryonic lung development, we generated P110*/+; *Dermo1-cre* mice by crossing P110*, a mouse line that allows conditional expression of a constitutively active form of p110α (Srinivasan et al., 2009), with *Dermo1-cre* mice (Figure 1A). *Dermo1* (also called *Twist2*) encodes a basic helix–loop–helix transcription factor and is highly expressed in mesodermal cells in mice (Figure 1B) (Fang et al., 2019). As the compound mutant mice expressing P110* in mesenchymal cells died before birth (E20.5) (Figure 1C), possibly due to overall defects of embryonic development, the lung phenotype analysis in this study is limited to the embryonic stages. Western blot analysis showed the activation of PI3K signaling in P110*/+; Dermo1-cre lungs (Figure 1D). By immunostaining, increased expression of p110α was found in mesenchymal cells with an antibody to P110α (Figure 1E). Surprisingly, phosphorylation of the protein kinase B (AKT), the direct downstream target of PI3K, was decreased in both the mesenchyme and epithelium in lungs from mutant mice (Figure 1D and Supplemental Figure S1), suggesting that the effects of PI3K signaling on embryonic lungs might not act through the well-established PI3K-AKT pathway.

**FIGURE 2:**
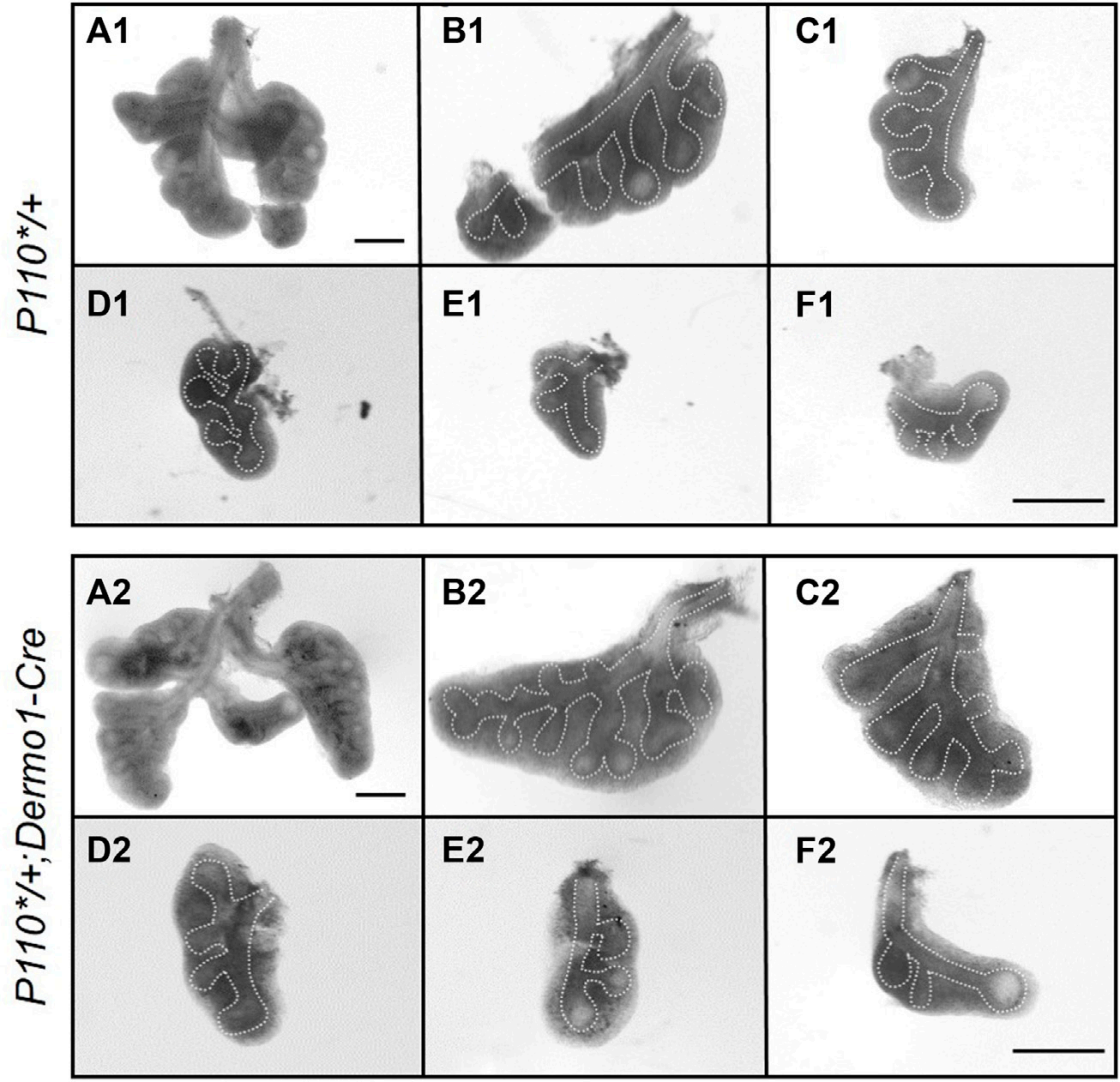
Activation of p110α promotes an early bifurcation of embryonic lungs. Whole lung and individual lobes of control and P110*/+; Dermo1-cre lungs. Scale bar: 50 μm.

To characterize the phenotypes, lungs from E12.5, E15.5, and E17.5 control and P110*/+; *Dermo1-cre* mice were isolated. Histologically, H&E stained sections of lungs from E12.5, E15.5, and E17.5 mutant embryos were nearly identical to the controls (Supplemental Figure S2). However, in E12.5 embryos, the overall size of the lungs of the mutant mice was consistently larger compared to that of controls. Although these lungs contained the same number of lobes, the size of the individual lobes was larger and contained more branches (Figure 2 and Supplemental Figure S3). Interestingly, this difference was decreased in E15.5 and E17.5 lungs (Supplemental Figure S4).

### Activation of p110α Promotes Branching in the Early Embryonic Stage Through *Isl1*-*Fgf10* Signaling

**FIGURE 3:**
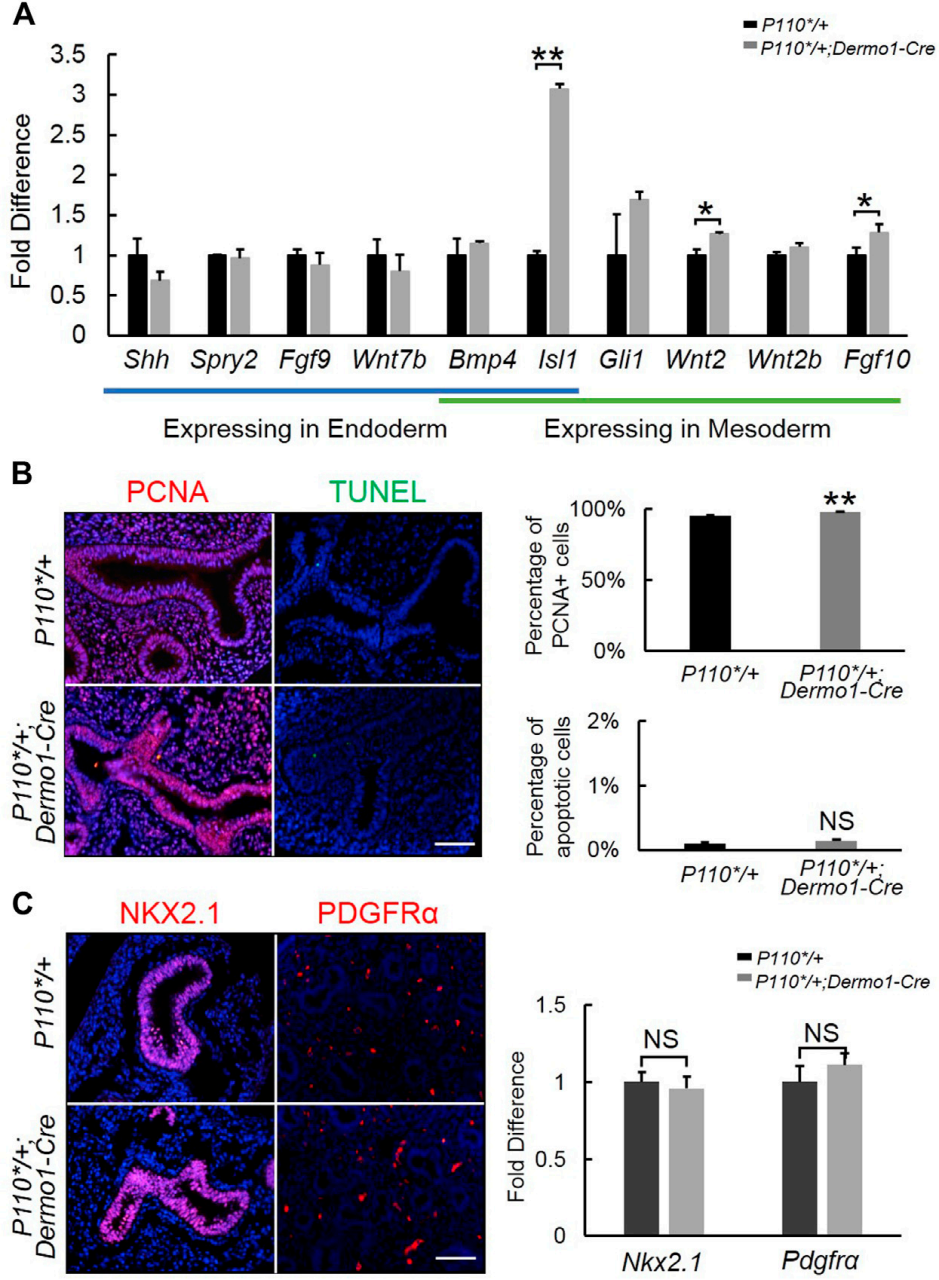
Detection of branching morphogenesis-related genes in early embryonic development. **(A)**. Real-time PCR analysis of branching morphogenesis-related gene expression in E12.5 control and P110*/+; Dermo1-cre lung. **(B)**. IF staining and cell counting for cell proliferation and apoptosis in E12.5 control and P110*/+; Dermo1-cre lung. **(C)**. IF staining analysis and real-time PCR analysis of *Nkx2.1* and *Pdgfrα* in E12.5 control and P110*/+; Dermo1-cre lungs. The bars represent the mean ± SD. *N* > 3. *p < 0.05, **p < 0.01. Scale bar: 50 μm.

To further examine the morphological effect of p110α activation on branching, we analyzed the lung tissues of E12.5 in the early stages of development. During branching morphogenesis, the epithelial airways undergo branching and outgrowth into the surrounding mesenchyme, which is regulated by signaling pathways such as *FGF10*, *Shh*, *BMP4*, the Wnt pathway, and others (Swarr and Morrisey, 2015). In the embryonic lungs of the mutant mice, no significant changes were observed in the ligands and mediators of the *Shh* and *BMP4* signaling pathways (Figure 3A). In contrast, increased expressions of *Fgf10* and its upstream regulator, *Isl1*, were observed (Figure 3A). The western blot analysis showed that the levels of FGF10 and ISL1 were higher in isolated E12.5 P110*/+; Dermo1-cre mesenchymal cells (Supplemental Figure S5) as compared to wild-type mesenchymal cells.

In E10.5–E12.5, *Fgf10* is strictly expressed in the distal mesenchymal cells where branching occurs and is essential to regulate airway epithelial cell proliferation (Yuan et al., 2018). To determine whether the increased branching morphogenesis in the lungs from mutant mice was due to increased cell proliferation or a decreased cell apoptosis, we examined *Pcna* by immunohistochemistry (Figure 3B) and real-time PCR (Supplemental Figure S6) for cell proliferation, and TUNEL staining (Figure 3B) and *Fn* expression (Supplemental Figure S6) for apoptosis. The results demonstrated that *Pcna* was significantly increased in E12.5, E15.5, and E17.5 P110*/+; *Dermo1-cre* lungs (Figure 3B, Supplemental Figure S7). Meanwhile, compared with controls, no change in cell apoptosis was found in the lungs of the E12.5, E15.5, and E17.5 P110*/+; *Dermo1-cre* mice (Figure 3B, Supplemental Figures S6,8).

To determine the impact of mesenchymal-specific p110α activation on early embryonic lung development, we analyzed the expression of NKX2.1 and PDGFR, markers of epithelial and mesenchymal progenitors, respectively. By immunohistochemistry and real-time PCR, no alteration was found in the lungs from E12.5 P110*/+; *Dermo1-cre* mice as compared to controls (Figure 3C).

### Reduced Club Cell Numbers in P110*/+; *Dermo1-Cre* Lungs

**FIGURE 4:**
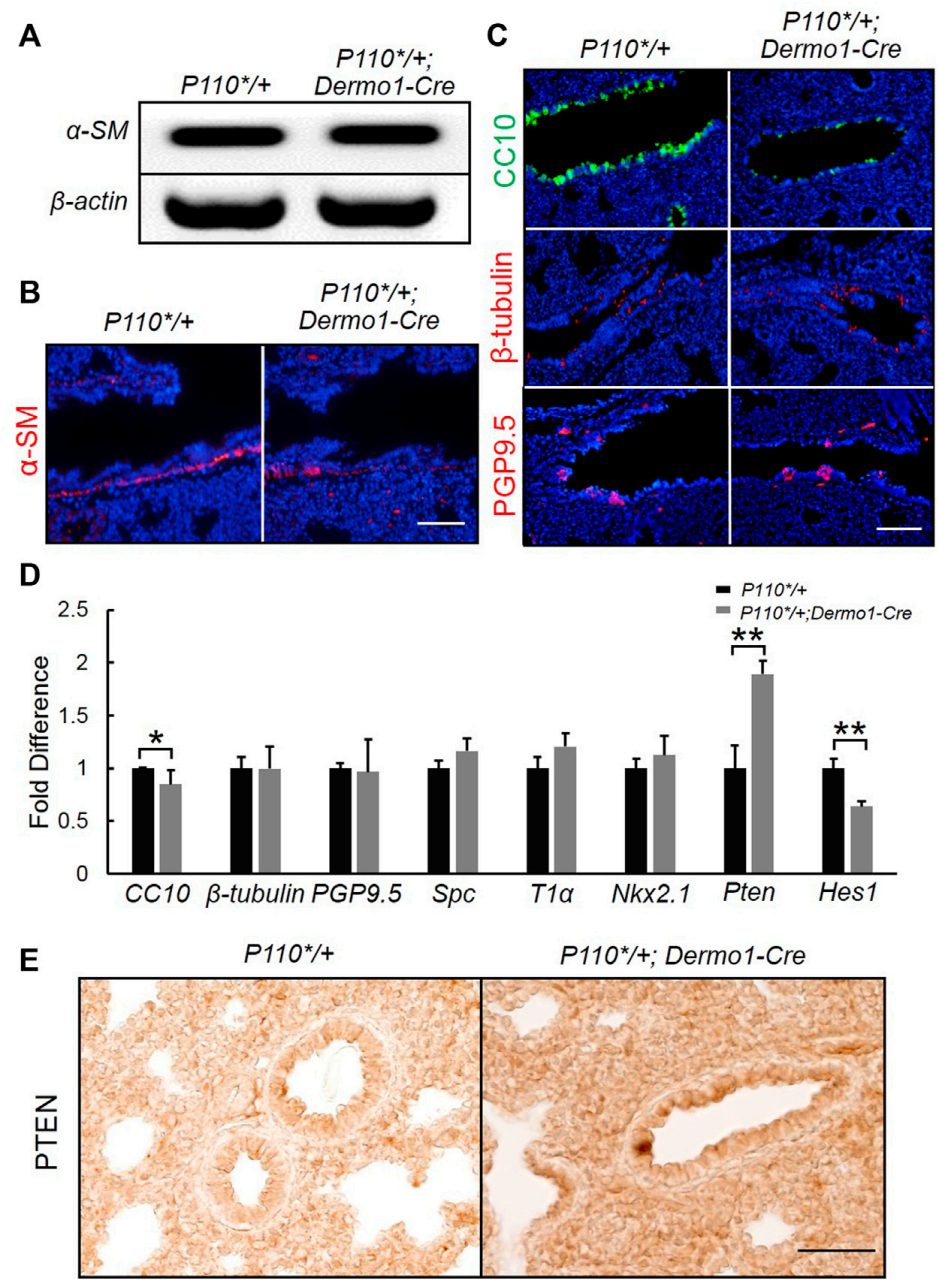
Activation of p110α resulted in a decrease in the number of club cells. Real-time PCR analysis **(A)** and IF staining analysis **(B)** of α-SM cells in E17.5 control and P110*/+; *Dermo1-cre* lungs. **(C)**. IF staining analysis of CC10, *ß*-tubulin, and PGP9.5 in E17.5 control and P110*/+; *Dermo1-cre* lungs. **(D)**. Real-time PCR analysis of *CC10, β-tubulin, PGP9.5, Spc, T1α, Nkx2.1, Pten,* and *Hes1* in E17.5 control and P110*/+; *Dermo1-cre* lungs. **(E)**. Immunolocalization analysis of PTEN in E17.5 control and P110*/+; Dermo1-cre lungs. The bars represent the mean ± SD. *N* > 3. *p < 0.05, **p < 0.01 Scale bar: 50 μm.

To study the potential impacts on the differentiation of mesenchymal cells in the later embryonic stage, we examined mesenchymal cell marker α-SMA (α-smooth muscle actin) in E17.5 lungs. Immunostaining and reverse transcription PCR (RT-PCR) analyses showed that α-SMA expression remained unaltered in P110*/+; *Dermo1-cre* lungs, suggesting that mesenchymal cell differentiation was not affected (Figure 4A,B). In the mouse lung, the epithelium is composed of functional compartments along a proximal–distal axis. The major differentiated cell types include club cells, ciliated cells, and neuroendocrine cells in the airways, and type I and type II cells in the alveoli. To investigate whether the mesenchymal activation of p110α signaling causes abnormalities in the composition of the lung epithelium, cell-specific markers for club, ciliated, neuroendocrine, and alveolar type I and type II cells were analyzed by immunostaining and real-time PCR. In the lungs from E17.5 P110*/+; *Dermo1-cre* mice, club cell numbers were significantly reduced as compared to control airways, as shown by CC10 staining and real-time PCR (Figure 4C,D). In contrast, the levels of Nkx2.1 in the lungs of E17.5 mice were not significantly altered (Figure 4D). PTEN, a multifunctional tumor suppressor, was initially identified as an epithelial cell-enriched phosphatase (Li et al., 1997). Previous studies have shown that the deletion of *Pten* in lung epithelial cells increases the number of club cells (Xing et al., 2010), and *Hes1* determined club cell differentiation (Ito et al., 2000). Consistent with the reduced number of club cells, in the lungs from E17.5 P110*/+; *Dermo1-cre* mice, the expression of *Pten* was significantly upregulated, whereas *Hes1* expression was reduced (Figure 4D). Interestingly, the overall expression of PTEN was increased in both the mesenchyme and epithelium in mutant lungs (Figure 4E). We propose that alteration in club cell numbers is due to the increased PTEN expression in the epithelium. However, the possibility that the PTEN signaling in the mesenchyme may participate in Hes1 regulation or the emergence of club cells requires further validation. By direct cell counting, the percentage of club cells over the total number of airway cells was reduced in mutant lungs, whereas the ratios of ciliated cells and neuroendocrine cells were not altered (Supplemental Figure S9). Antibodies to T1α and SPC were used as an alveolar type I and type II cell markers, respectively. Compared with control lungs, the expression of T1α and SPC were not altered in mutant lungs (Figure 4D; Supplemental Figure S10). Therefore, the activation of p110α in the mesenchyme affects club cell differentiation, but not ciliated, neuroendocrine, and alveolar cells.

### Activation of PI3K in Mesenchymal Cells Blocks Differentiation of SSEA1^+^ Progenitor Cells Into Club Cells

**FIGURE 5:**
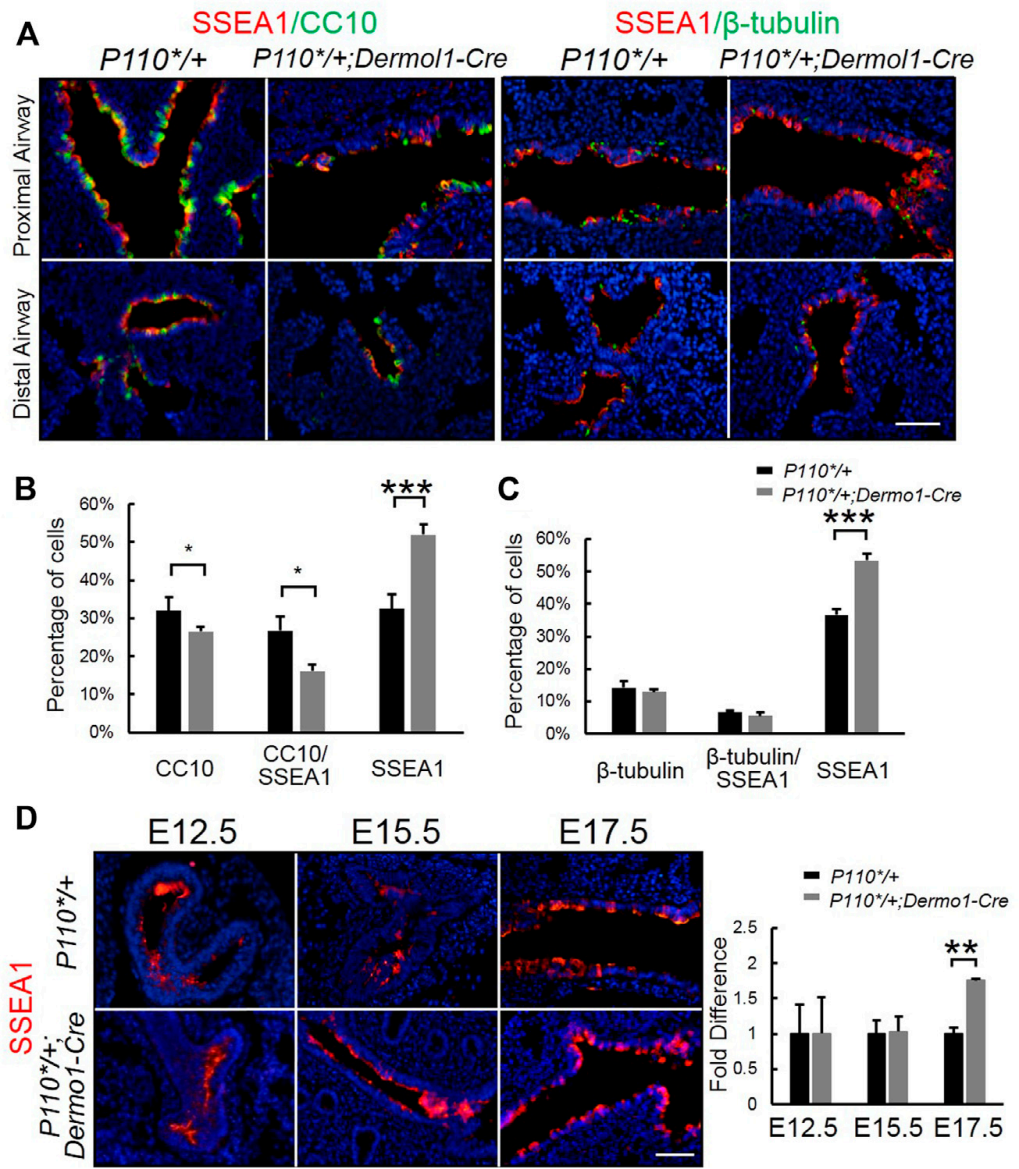
Activation of p110α inhibits the differentiation of epithelial progenitor cells. **(A)**. Double IF staining analysis of CC10, SSEA1, and *ß*-tubulin in E17.5 control and P110*/+; *Dermo1-cre* lungs. **(B,C)**. The percentages of CC10, CC10/SSEA1, *ß*-tubulin, *ß*-tubulin/SSEA1, and SSEA1 cells in total E17.5 airway cells were counted on multiple random fields. **(D)**. IF staining analysis and real-time PCR analysis of SSEA1 in E12.5, E15.5, and E17.5 control and P110*/+; *Dermo1-cre* lungs. The bars represent the mean ± SD. *N* > 3. *p < 0.05, **p < 0.01***p < 0.001 Scale bar: 50 μm.

SSEA1 (stage-specific embryonic antigen-1) is a marker of mouse embryonic stem (ES) cells, and its expression is turned off upon the differentiation of ES cells (Henderson et al., 2002). SSEA1 is specifically expressed in airway epithelial progenitors (Xing et al., 2010). To identify whether the reduced number of club cells in the mutant airway was due to impaired progenitor differentiation, we used anti-SSEA1 and anti-CC10 or anti-*β*-tubulin antibodies to distinguish various airway epithelial populations at distinct differentiation status in E17.5 lungs. These included SSEA1-positive (SSEA1^+^) progenitor cells; SSEA1-and CC10-positive differentiating club cells (SSEA1^+^/CC10^+^); CC10-positive (CC10^+^) terminally differentiated club cells; SSEA1-and *β*-tubulin-positive (SSEA1^+^/*β*-tubulin^+^) differentiating ciliated cells; and *β*-tubulin-positive (*β*-tubulin^+^) terminally differentiated ciliated cells (Figure 5A). Compared with the lungs of control mice, an increased number of SSEA1^+^ cells and reduced number of CC10^+^ cells were found to be localized along the airways in the mutant mice, whereas the percentage of *ß*-tubulin-positive cells remained unchanged (Figure 5B,C). The levels and patterns of SSEA1 expression were not significantly different between the mutant and control lungs at E12.5 and E15.5. However, at E17.5, SSEA1 levels were largely increased in the mutant lungs (Figure 5D). These results show that the mesenchymal activation of p110α affects bronchiolar epithelial progenitor cell differentiation.

### Activation of PI3K in Mesenchymal Cells Promotes Expansion of SOX9 but not SOX2

**FIGURE 6:**
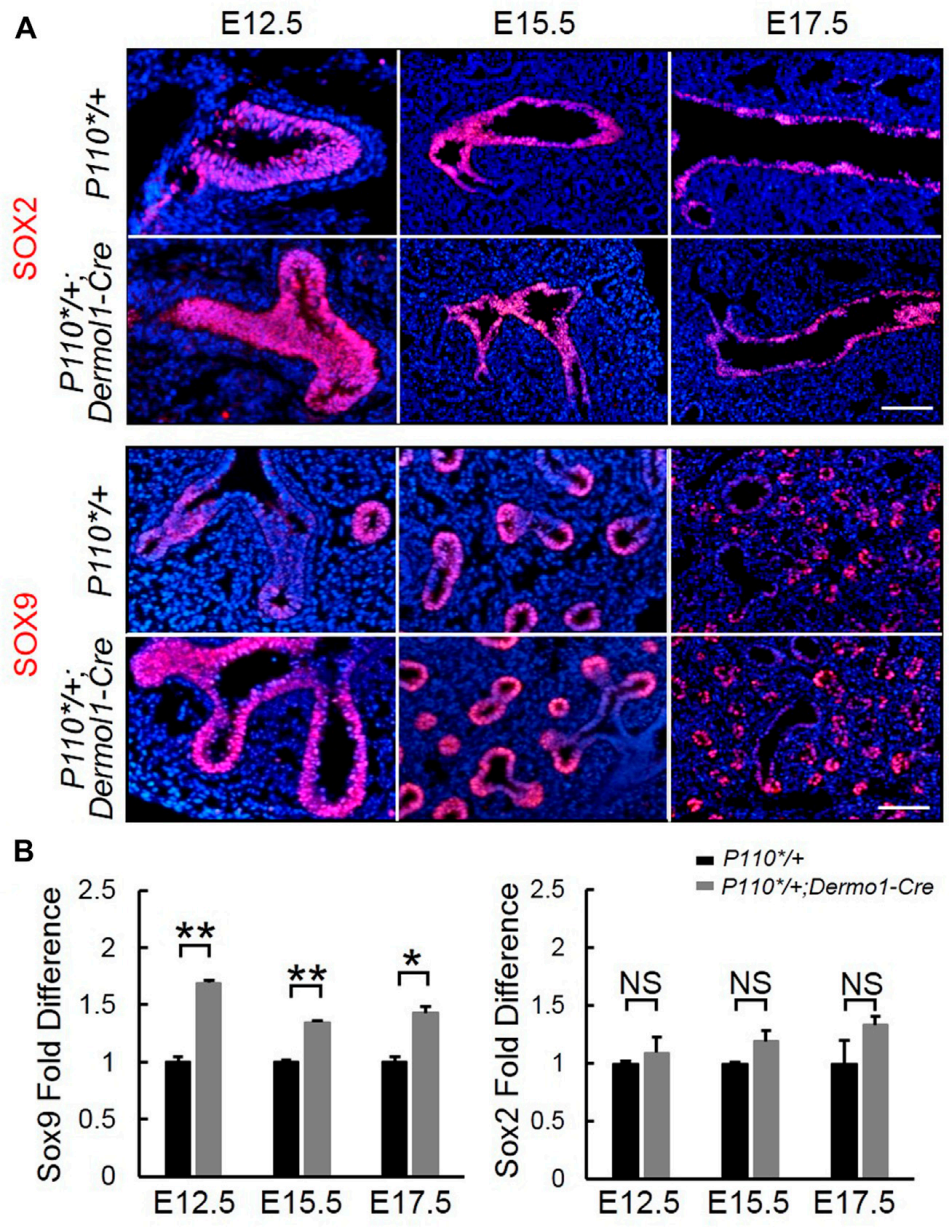
Mesenchymal activation of p110α affects the SOX9 expression in the distal epithelium. IF staining analyses **(A)** and real-time PCR analyses **(B)** of *Sox2 and Sox9* expression in E12.5, E15.5, and E17.5 control and P110*/+; *Dermo1-cre* lungs. The bars represent the mean ± SD. *N* > 3. *p < 0.05, **p < 0.01 Scale bar: 50 μm.

Sox9 and Sox2 regulate the branching morphogenesis and epithelial cell differentiation along a proximal–distal axis during lung development (Danopoulos et al., 2018). To determine whether mesenchymal-activated PI3K signaling disrupted airway branching through *Sox9* and (or) *Sox2*, we analyzed *Sox9* and *Sox2* expression during lung development. As shown in Figure 6A, SOX9 was highly expressed in the distal epithelium, whereas SOX2 was restricted to the proximal epithelial in the lungs from both control and P110*/+; *Dermo1-cre* mice. Consistent with the increased branching, a larger number of SOX9-positive distal airway tips were observed in the mutant lungs (Figure 6A). In addition, *Sox9* transcript levels were also significantly increased in E12.5, E15.5, and E17.5 P110*/+; *Dermo1-cre* lungs (Figure 6B). In contrast to *Sox9*, the expression of *Sox2* in the lungs of mutant mice appeared unchanged (Figure 6B).

**FIGURE 7:**
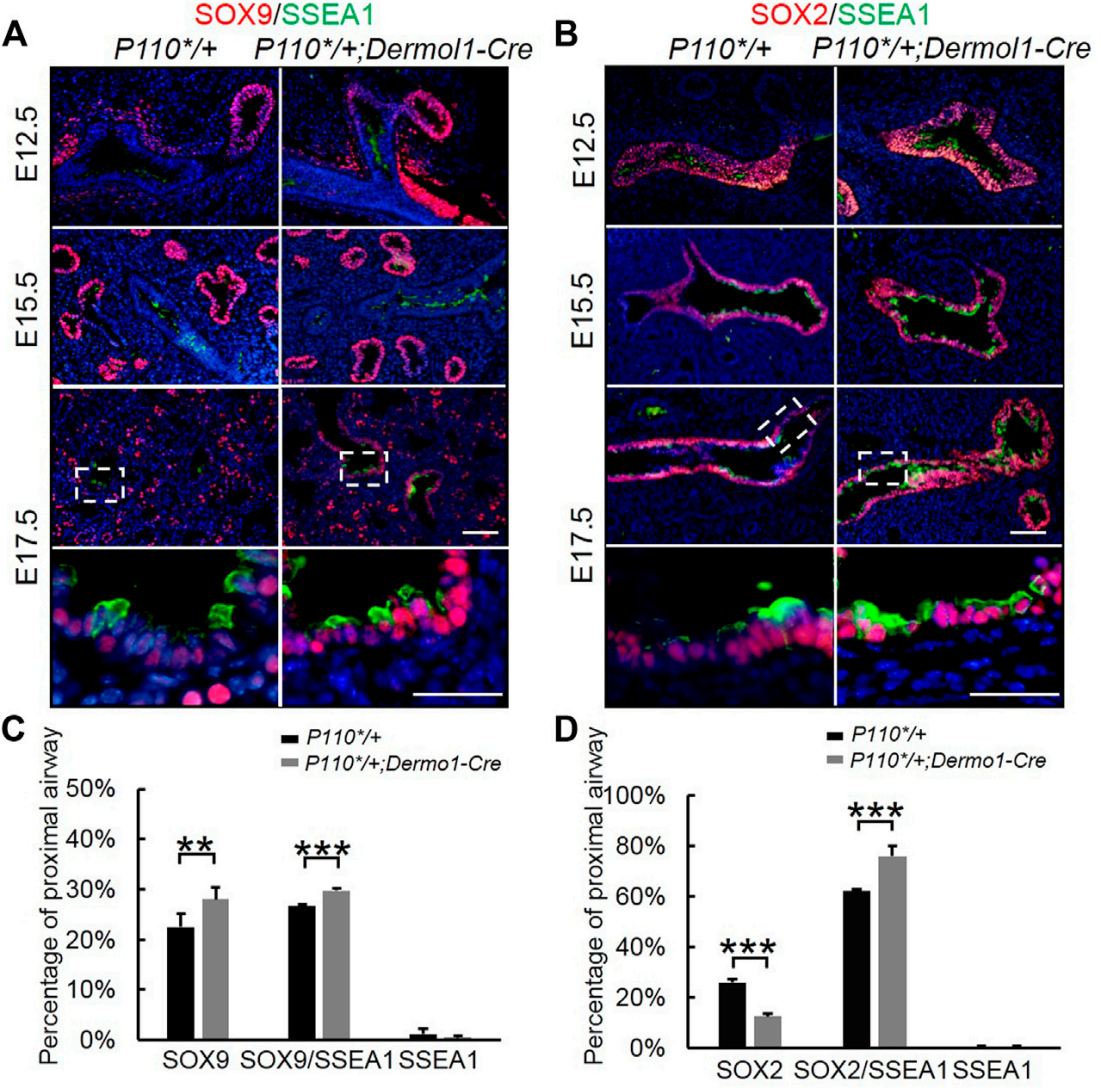
Analyses of epithelial progenitor cell differentiation. **(A,B)**. Double IF staining analyses of SOX2, SOX9, and SSEA1 expression in E17.5 control and P110*/+; *Dermo1-cre* lungs. **(C)**. Percentages of SOX9, SOX9/SSEA1, and SSEA1 cells in the total E17.5 distal airway cells were counted on multiple random fields. *N* > 3. **(D)**. Percentages of SOX2, SOX2/SSEA1, and SSEA1 cells in the total E17.5 proximal airway cells were counted on multiple random fields. The bars represent the mean ± SD. *N* > 3. **p < 0.01***p < 0.001. Scale bar: 50 μm.

Initial assessment of the E17.5 P110*/+; *Dermo1-cre* lungs showed that the airway epithelial cells were arrested at the SSEA1^+^ progenitor state (Figure 5). To identify the distribution of SSEA1^+^ cells along the distal and proximal airways, antibodies to SOX9 and SOX2 were used to label SSEA1^+^ cells, respectively (Figure 7). In the lungs of E12.5 and E15.5 mice, the SSEA1^+^ cells in the proximal airway were labeled by SOX2, whereas in E17.5 lungs, the SSEA1^+^ cells were labeled by SOX2 and SOX9 in the proximal and distal airways, respectively (Figure 7A,B). The ratio of each group of cells over the total number of distal or proximal airway cells was calculated by cell counting. Consistent with the increased *Sox9* expression and branching, the percentage of SOX9^+^ cells (57.78%), including SOX9^+^/SSEA1^+^ cells (29.76%) and SOX9^+^/SSEA1^-^ cells (28.02%), was significantly higher in the lungs from the mutant mice than in those control animals (49.21%) (Figure 7C). Surprisingly, although the total percentage of SOX2^+^ cells was not altered (88.31% in mutant vs. 88.08% in control lungs), around 13% more SOX2^+^/SSEA1^+^ cells were detected in the mutant lungs than in those from control lungs where SOX2-labeled SSEA1^-^ cells in the mutant lungs was 13.44% less than in the control lungs (Figure 7D). The ratios of SSEA1^+^ cells in the distal (31.1% in mutant vs. 27.7% in control lungs) and proximal airways (76.4% in mutant vs. 62.6% in control lungs) were increased, suggesting that the blockage of SSEA1^+^ progenitor cell differentiation was not restricted to a certain region(s).

## Discussion

**FIGURE 8:**
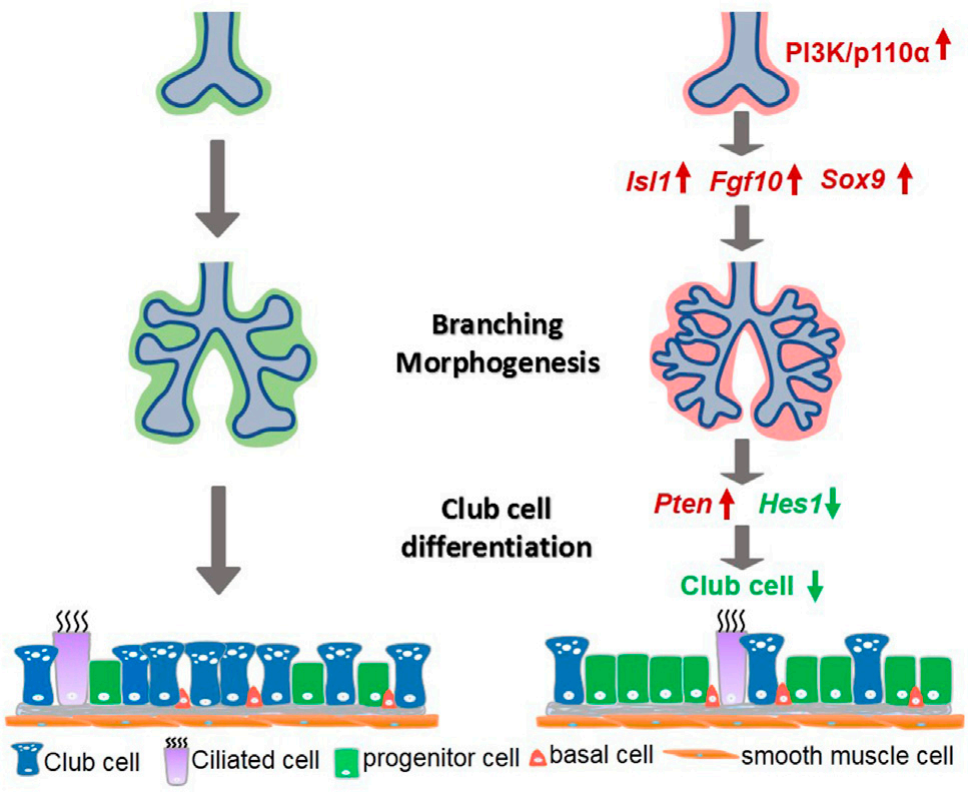
A simplified model illustrating PI3K/p110α signaling in mesenchymal lung development. Mesenchymal activation of PI3K/p110α promoted branching morphogenesis through the *Isl1*–*Fgf10*–*Sox9* pathway, and regulated club cell differentiation by the *Pten*-*Hes1* pathway.

The purpose of this study was to investigate the accurate role of mesenchymal-specific PI3K in lung development. The choice of PI3K signaling was based on the diverse outcomes it generates in the development of branched tissues, including proliferation, motility, growth, survival, and cell death (Zhu and Nelson, 2012). The mammalian lung represents an attractive model as it consists of millions of airway branches and more than 40 distinct specialized cell varieties. We found that the mesodermal activation of p110α resulted in abnormal lung morphogenesis characterized by increased branching and cell proliferation in the E12.5 lungs. As development progressed, these phenotypes evolved into enlarged lungs in E12.5 and later stages. An examination of cell differentiation revealed a marked reduction of club cells in the p110α mutant lungs. These results provide novel evidence that signaling through PI3K-p110α plays a role as a mediator of epithelial–mesenchymal interactions in branching morphogenesis and cell differentiation, potentially mediated via the Isl1–Fgf10–Sox9 and Pten–Hes1 pathway networks, respectively (Figure 8).

The PI3K signaling pathway is complex. It is generally thought that AKT is a common downstream target of PI3K signaling and is considered a master regulator mediating cell proliferation, motility, and survival (Liu et al., 2014). In the mouse lung, the level of AKT phosphorylation is the highest at E12 and gradually decreases thereafter (Wang et al., 2005), implying that AKT regulates an early stage of lung development (E9.5–E12.5). Surprisingly, examination of AKT phosphorylation in p110α mutant lungs showed a significant reduction. This may be explained by several potential mechanisms. First, it is well established that PTEN serves as a negative regulator of PI3K signaling by dephosphorylating PIP3. The current study revealed that the activation of the PI3K pathway via p110α overexpression resulted in an increased PTEN expression. This indicates that a negative feedback mechanism may exist in the embryonic lung to counteract the overactivation of the PI3K pathway via PTEN. Since PTEN inhibits AKT phosphorylation, it is likely that the PTEN activation in response to p110α overexpression eventually led to decreased AKT phosphorylation. Alternatively, other classes of PI3K enzymes or other class I catalytic isoforms may be involved in the regulation of AKT phosphorylation in the lung mesoderm. Overexpression of the p110 subunit alone may interfere with the expression or function of other PI3k subunits and consequently lead to reduced AKT activation. The third possibility may involve other upstream regulators of AKT phosphorylation. Previous studies have highlighted a diverse group of tyrosine (AckI/TNK2, Src, and PTK6) and serine threonine (TBKI, IKBKE, and DNAPKcs) kinases that activate AKT directly to promote its pro-proliferative signaling functions (Mahajan and Mahajan, 2012). Overexpression of p110a may disrupt the action of the latter AKT regulators. Further examination of the latter possibilities may reveal novel mechanisms of PI3K signaling which is essential to many key cellular activities. Although it is difficult to identify the reduction of AKT phosphorylation present in the epithelial or mesenchymal compartments, it appears that there may be other upstream regulators of AKT phosphorylation.

PTEN acts as a principal negative regulator to modulate the effects of the PI3K pathway during embryonic development. Homozygous PTEN deletion mice die between E6.5 and E9.5 (Cristofano et al., 1998). By crossing *PTEN* ^*fl/fl*^ mice with *Nkx2.1-cre* mice, epithelial-specific ablation of PTEN leads to club cell hyperplasia but does not disrupt branching morphogenesis (Tiozzo et al., 2009; Xing et al., 2010). Thus, the upregulated branching morphogenesis in p110α mutant lungs may not be due to the increased *Pten* expression. The findings in the present study are consistent with the interpretation that *Pten* serves as a key factor for club cell differentiation.

*Isl1* encodes a transcription factor associated with multipotency of human cardiac progenitors (Cai et al., 2003; Bu et al., 2009). *Isl1* null mice die at E10.5 due to severe heart problems (Ahlgren et al., 1997). In *Isl1−/−* mice, the hearts no longer express certain Bmp or Wnt family members, FGF8 or FGF10. In addition, a novel enhancer, which contains a highly conserved ISL1 consensus binding site, is identified within the *FGF10* first intron (Golzio et al., 2012). In the developing lung, both the epithelium and mesenchyme of the trachea express *Isl1* at E11.5, and *Isl1* expression remains high only in the ventral epithelium and mesenchyme of the trachea but low in the epithelium of the budding tips at E13.5. At E14.5, *Isl1* is expressed in the mesothelium and becomes undetectable in the epithelium (Chang et al., 2013). In the present studies, the expression of *Isl1* and *Fgf10* was stimulated, and the branch number and cell proliferation were also increased in E12.5 p110α mesenchymal activation lungs. The direct regulation of FGF10 by ISL1 has previously been demonstrated (Golzio et al., 2012). Thus, the current data support the notion that, during branching morphogenesis, PI3K/p110α signaling regulates the expression of *Isl1* and the downstream target, *Fgf10*, in mesenchymal cells around the distal lung epithelial tips, which is then transmitted to *Fgfr2*, the *Fgf10* receptor, in the developing endoderm. However, precisely how the p110α-ISL1–FGF10 pathway mediates the p110α functions during lung branching morphogenesis remains to be further elucidated.

Sox family members have been shown to mediate the specification and differentiation of a variety of cell types. Among them, the expression of SOX2 and SOX9 marks distinct cell lineages along the lung endoderm proximal–distal axis (Herriges and Morrisey, 2014). The SOX9^+^ progenitors located in the distal tips of the endoderm receive the signals and promote branching (Chang et al., 2013). FGF10 has been identified to be the major mesenchymal signal that promotes distal airway branching. As the branches grow distally, the proximal airway cells downregulate *Sox9* expression and upregulate *Sox2* expression and, eventually differentiate into the club, ciliated, and secretory cells. In the current study, we demonstrated that a mesenchymal-specific overexpression of p110α resulted in increased FGF10 and SOX9 levels, accompanied by increased airway branching in E12.5 lungs. This indicates that the activation of the PI3K pathway increases FGF10 expression which promotes expansion and branching of Sox9+ distal airway epithelial progenitors. In contrast, neither Sox2 expression nor SOX2+ cell numbers were altered by p110α overexpression.

Interestingly, overexpression of p110α increased the levels of embryonic stem cell marker SSEA1 in both the SOX9+ and SOX2+ populations, as represented by an increased number of SOX9+/SSEA1+ and SOX2+/SSEA1+ cells in the p110α mutant lungs. Since SSEA1 expression is associated with the stem vs. differentiation status of ES cells (Henderson et al., 2002), the PI3K signaling pathway appears to play an important role in cell fate determination of both proximal and distal airway progenitors. This is supported by the fact that there is a significant increase of SOX2+/SSEA1+ cells and reduced club cell differentiation in p110α mutant lungs.

In sum, our findings suggest that, during the embryonic lung branching morphogenesis and cell differentiation, PI3K signaling via p110α plays important roles in mediating mesenchymal–epithelial interactions through pathways that involve the *Isl1*–*Fgf10*–*Sox9* and *Pten*–*Hes1* networks.

## Data Availability

The original contributions presented in the study are included in the article/Supplementary Material, further inquiries can be directed to the corresponding author.
