# Supplementary material for: Activation of PI3K/p110α in the Lung Mesenchyme Affects Branching Morphogenesis and Club Cell Differentiation
Source: Front Cell Dev Biol. 2022 May 23;10:880206. doi: 10.3389/fcell.2022.880206 (PMC9168599; doi:10.3389/fcell.2022.880206)
Supplement: Supplementary file 1 [file DataSheet1.docx]

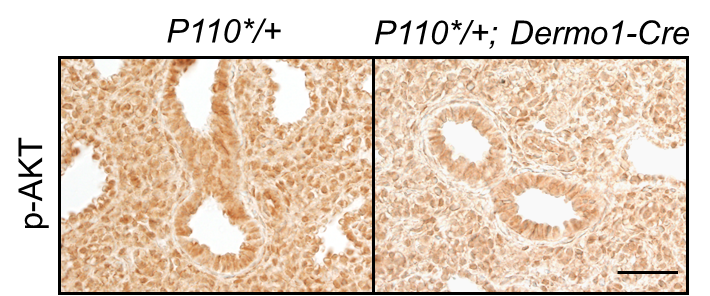


**Supplemental Figure 1. Overexpression of p110α reduces AKT phosphorylation in both mutant mesenchymal and epithelial cells.**

Immunostaining of p-AKT in E17.5 control and *P110*/+; Dermo1-cre* lungs. Scale bar: 50μm.


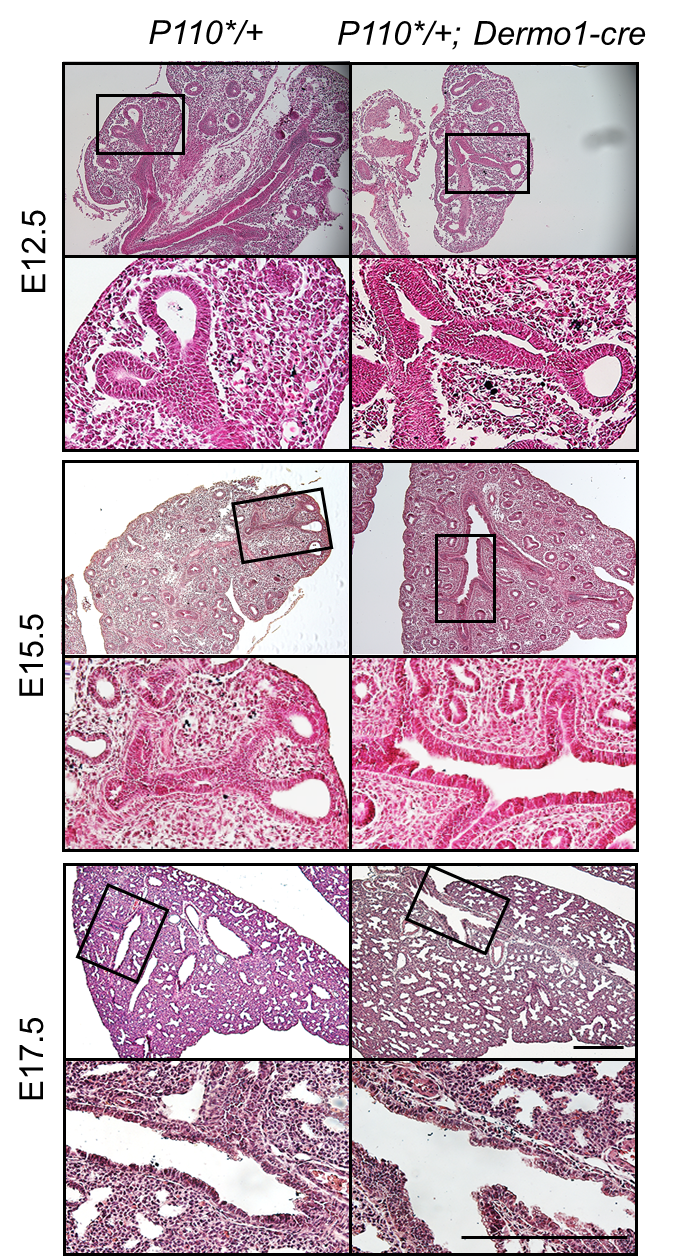


**Supplemental Figure 2. Histology of mouse embryonic lungs.**

H&E sections of control and *P110*/+; Dermo1-cre* lungs at E12.5, E15.5 and E17.5. Scale bar: 50μm.


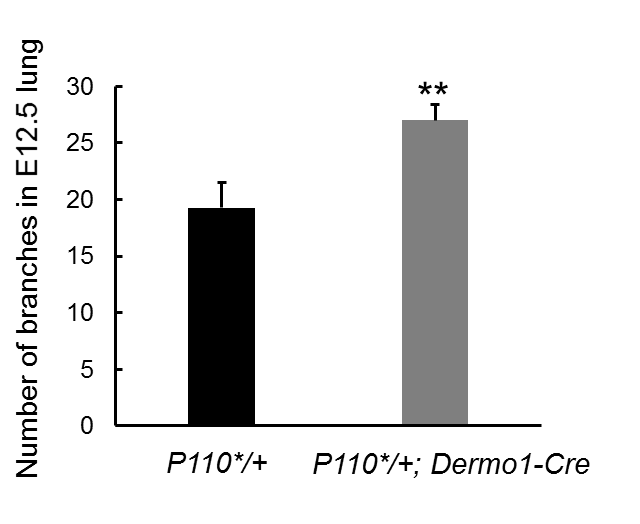


**Supplemental Figure 3. Overexpression of p110α promotes branching morphogenesis in E12.5 *P110*/+; Dermo1-cre* lungs.**

Quantification of pulmonary branches in E12.5 control and *P110*/+; Dermo1-cre* lung. The bars represent the mean ± SD. N>3. ***P*<0.01.


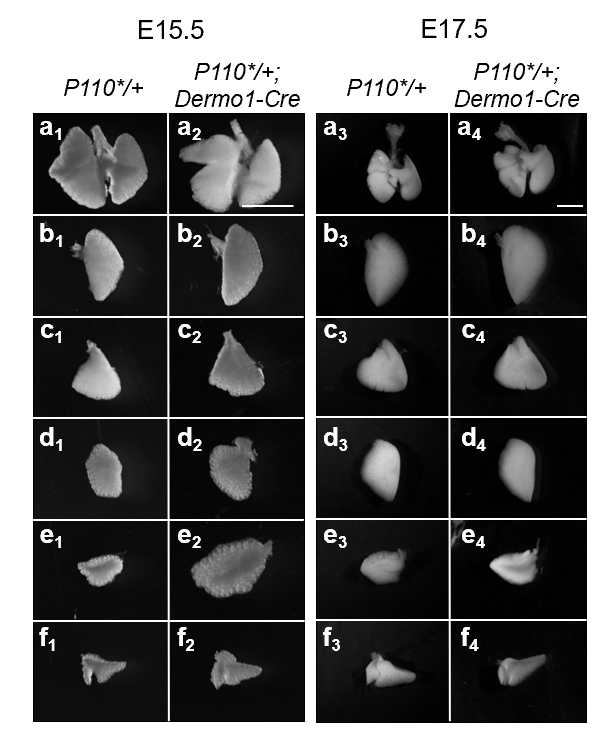


**Supplemental Figure 4.** **Gross morphology of embryonic lungs.**

Whole lung and individual lobes of control and *P110*/+; Dermo1-cre* lung. **a1-f1.** E15.5 control lung. **a2-f2.** E15.5 *P110*/+; Dermo1-cre* lung. **a3-f3.** E17.5 control lung. **a4-f4.** E17.5 *P110*/+; Dermo1-cre* lung. Scale bar: 200μm.


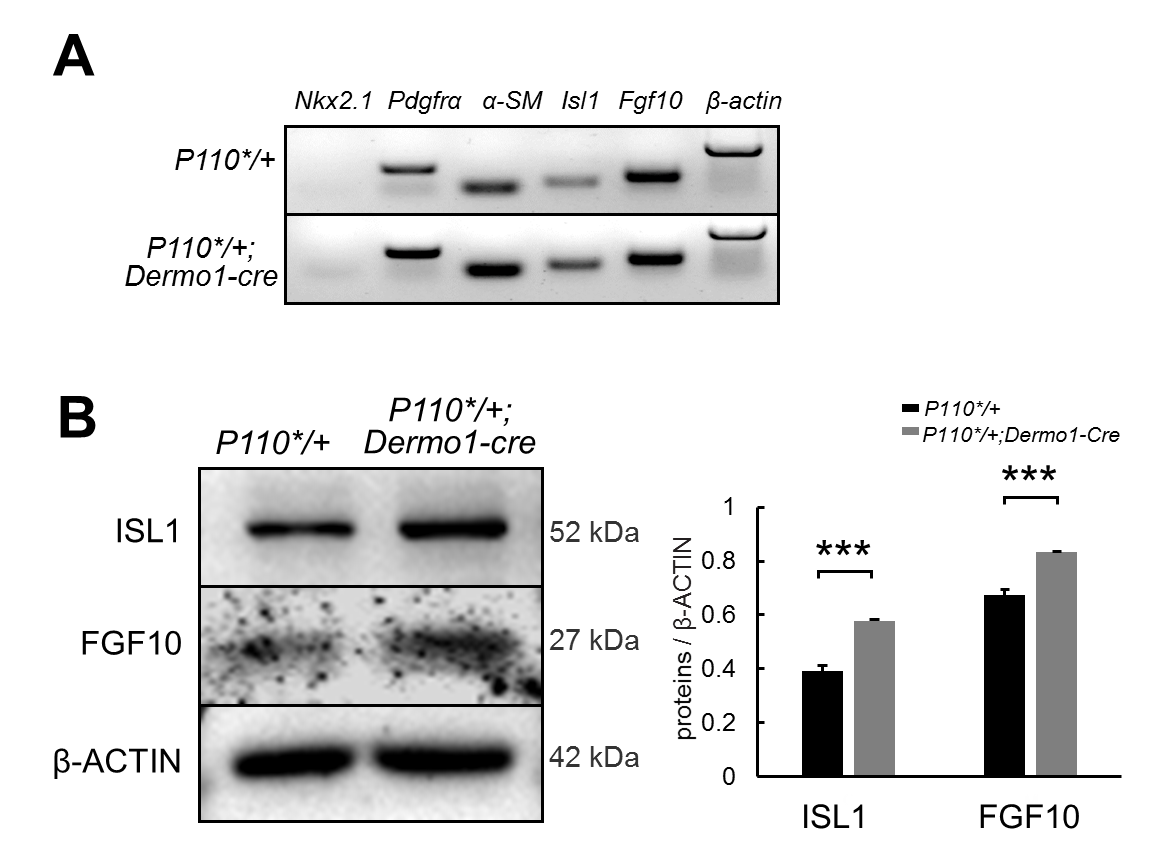


**Supplemental Figure 5. Activation of p110α promotes the expression of FGF10 and ISL1 in E12.5 *P110*/+; Dermo1-cre* lung.**

**(A)**. The expression of mesenchymal cell markers (PDGFα and α-SM) and epithelial cell marker (Nkx2.1) in isolated mesenchymal cells from E12.5 control and *P110*/+; Dermo1-cre* lungs was analyzed by RT-PCR. **(B)**. Western blot analyses of ISL1 and FGF10 expression in isolated mesenchymal cells from E12.5 control and *P110*/+; Dermo1-cre* lungs. ****P*< 0.001.


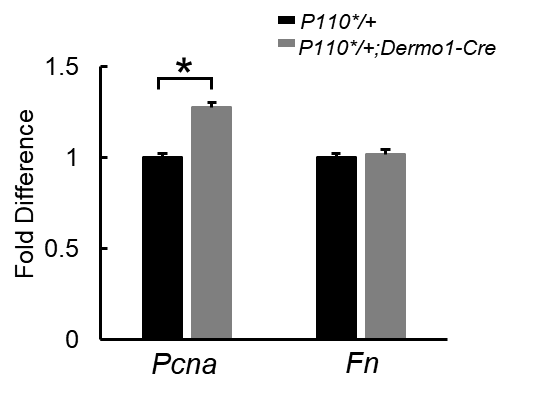


**Supplemental Figure 6. Activation of p110α promotes cell proliferation in E12.5.**

Real-time PCR analyses of *Pcna* and *Fn* expression in E12.5 control and *P110*/+; Dermo1-cre* lung. The bars represent the mean ± SD. N>3. **P*<0.05. Scale bar: 50μm.


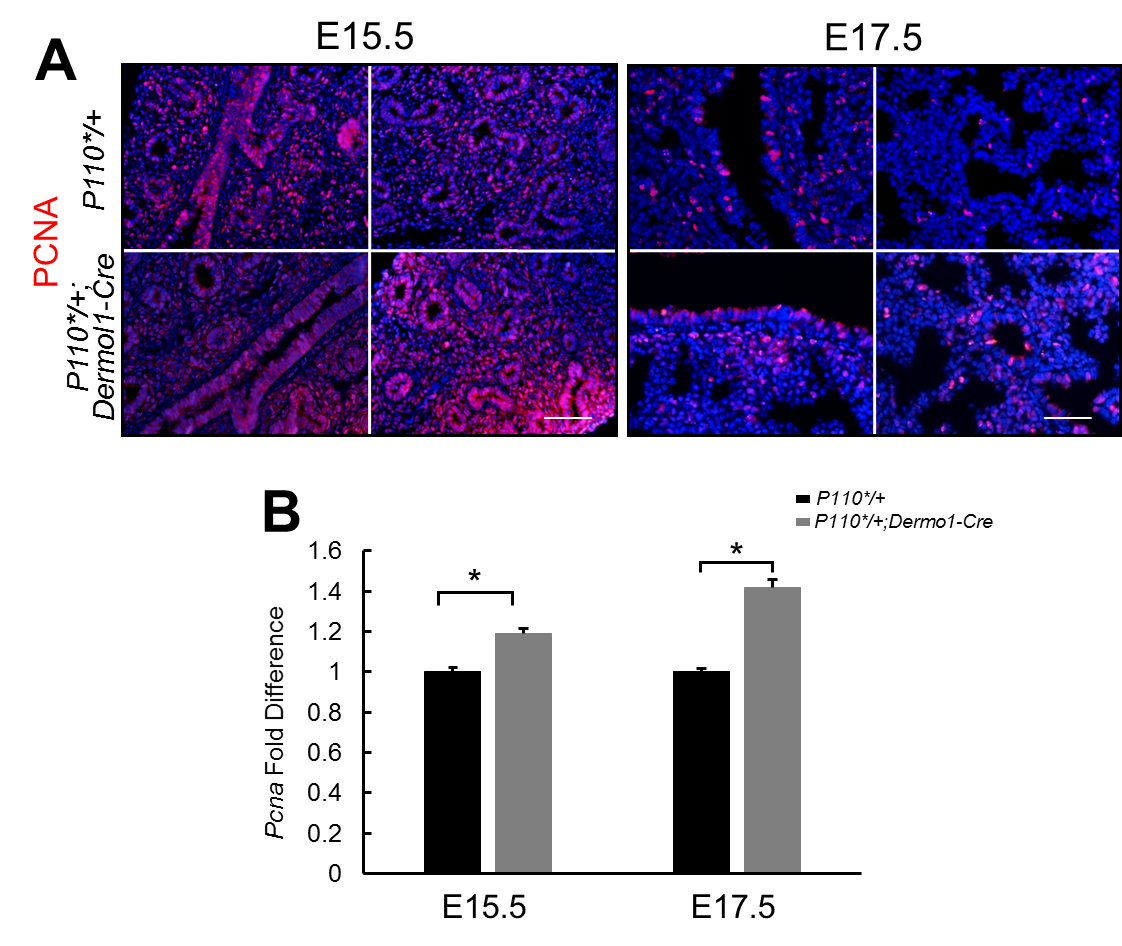


**Supplemental Figure 7. Activation of p110α promotes cell proliferation in E15.5 and E17.5 lungs.**

**A.** Immunofluorescence staining of PCNA in E15.5 and E17.5 control and *P110*/+; Dermo1-cre* lungs. **B.** Real-time PCR analysis of *Pcna* expression in E15.5 and E17.5 control and *P110*/+; Dermo1-cre* lung. The bars represent the mean ± SD. N>3. **P*<0.05, Scale bar: 50μm.


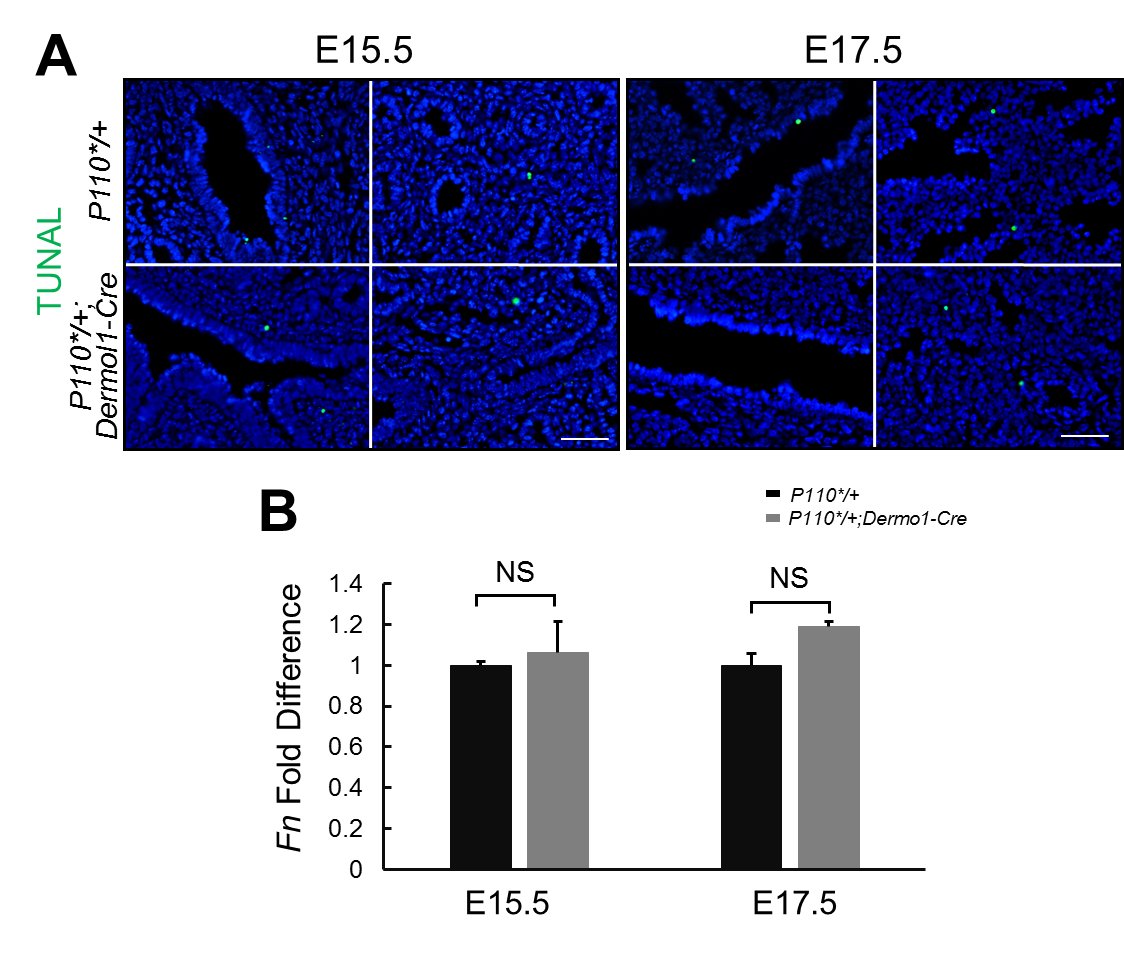


**Supplemental Figure 8. Activation of p110α does not affect cell apoptosis in E15.5 and E17.5 lungs.**

**A.** TUNAL analysis for cell apoptosis in E15.5 and E17.5 control and *P110*/+; Dermo1-cre* lungs. **B.** Real-time PCR analysis of *Fn* in E15.5 and E17.5 control and *P110*/+; Dermo1-cre* lungs. The bar represents the mean ± SD. N>3. *NS*<0.05, Scale bar: 50μm.


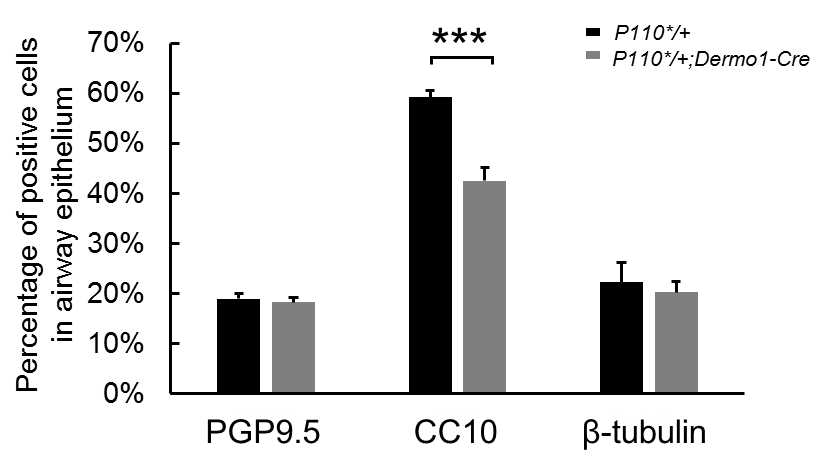


**Supplemental Figure 9. Overexpression of p110α does not affect ciliated cell and neuroendocrine cell differentiation.**

Percentage of PGP9.5, CC10 and β-tubulin positive cells in total E17.5 airway epithelium cells were counted on multiple random fields. The bars represent the mean ± SD. N>3. ****P*<0.001.


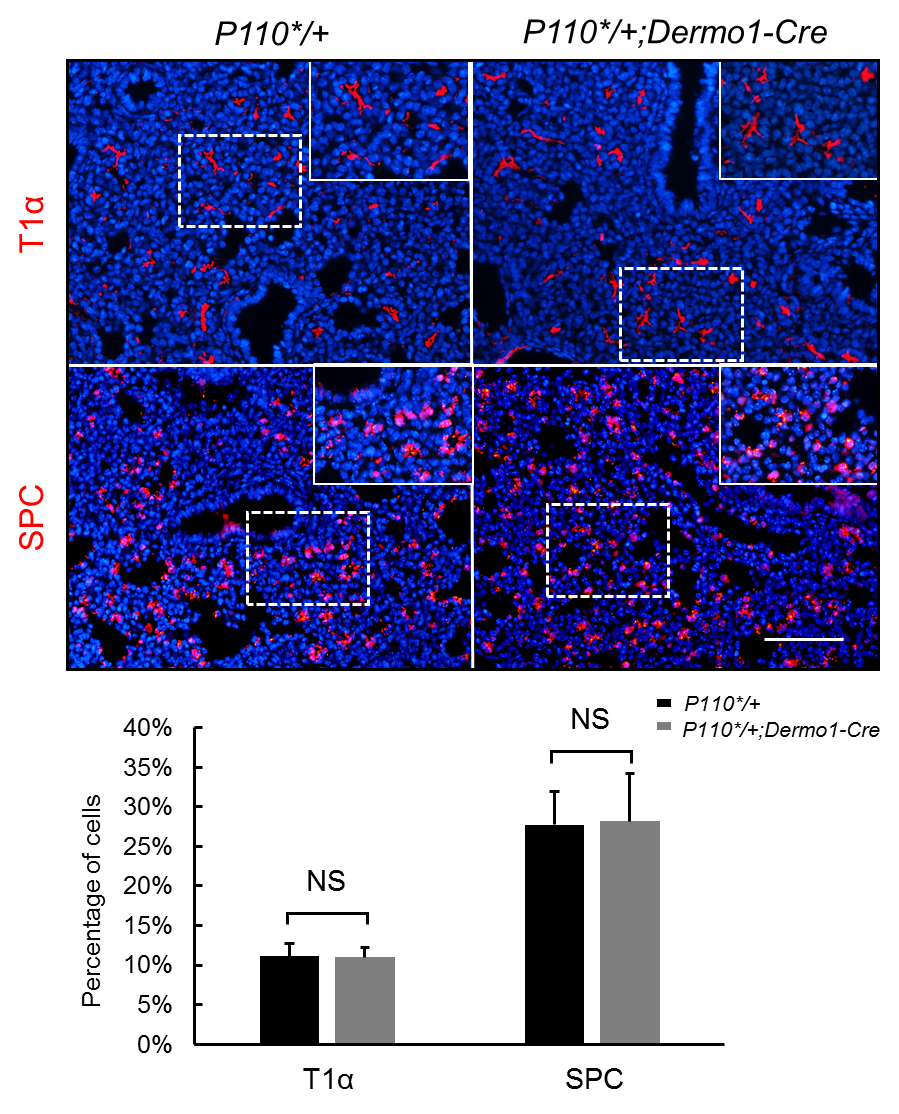


**Supplemental Figure 10. Activation of p110α in mesenchyme does not affect alveolar type Ⅰ cells and type Ⅱ cells differentiation.**

**A.** Immunofluorescence staining of T1α and SPC in E15.5 and E17.5 control and *P110*/+; Dermo1-cre* lungs. Boxed areas are enlarged and placed on top left. **B.** Percentage of T1α and SPC positive cells in total mesenchymal cells were counted on multiple random fields. The bar represents the mean ± SD. N>3. *NS*<0.05, Scale bar: 50μm.

| **Name** | **Host species** | **Source** | **Identifer** | **Application** | |  |
| --- | --- | --- | --- | --- | --- | --- |
| NKX2.1 | Rabbit | Cell signaling Technology | 12373 | | IF | |
| PDGFRα | Rabbit | Cell signaling Technology | 3174 | | IF | |
| PCNA | Mouse | Cell signaling Technology | 2586 | | IF | |
| SSEA1 | Mouse | Millipore Merck | MAB4301 | | IF | |
| SOX2 | Rabbit | Millipore Merck | ab5603 | | IF | |
| SOX9 | Rabbit | Cell signaling Technology | 82630 | | IF | |
| CC10 | Goat | Santa Cruz | sc-9772 | | IF | |
| β-tubulin Ⅳ | Mouse | Biogenex | MU178-UC | | IF | |
| KI67 | Mouse | Cell signaling Technology | 9449 | | IF | |
| GFP | Chicken | Abcam | Ab13970 | | IF | |
| α-SMA | Rabbit | Abcam | ab5694 | | IF | |
| T1α | Syrian hamster | HDSB | 8.1.1 | | IF | |
| SPC | Mouse | Santa Cruz | sc-518029 | | IF | |
| PI3K-P110α | Rabbit | Cell signaling Technology | 4249 | | WB/IHC | |
| P-Akt（Ser473） | Rabbit | Cell signaling Technology | 4060 | | WB/IHC | |
| PTEN | Rabbit | Cell signaling Technology | 9559 | | IHC | |
| PGP9.5 | Rabbit | Cell signaling Technology | 13179 | | IF | |
| ISL1 | Rabbit | Abcam | ab20670 | | WB | |
| FGF10 | Rabbit | Abclonal | A1201 | | WB | |
| β-ACTIN | Mouse | Qualit Yard | QYA10733A | | WB | |

**Supplemental Table 1. Primary antibody list**

The primary antibodies used in this study were listed.

| **Primer** | **Sequence (5’-3’)** |
| --- | --- |
| *Fgf10*-F | TTTGGTGTCTTCGTTCCCTGT |
| *Fgf10*-R | TAGCTCCGCACATGCCTTC |
| *Fgf9*-F | ATGGCTCCCTTAGGTGAAGTT |
| *Fgf9*-R | TCATTTAGCAACACCGGACTG |
| *Wnt7b*-F | TTTGGCGTCCTCTACGTGAAG |
| *Wnt7b*-R | CCCCGATCACAATGATGGCA |
| *Shh*-F | AAAGCTGACCCCTTTAGCCTA |
| *Shh*-R | TTCGGAGTTTCTTGTGATCTTCC |
| *Isl1*-F | CAGTCCCAGAGTCATCCGAGT |
| *Isl1*-R | TGGGTTAGCAGTTTTGTCGTT |
| *Nkx2.1*-F | AGGACACCATGCGGAACAG |
| *Nkx2.1*-R | CCATGCCGCTCATATTCATGC |
| *Pdgfra*-F | TGCGGGTGGACTCTGATAATGC |
| *Pdgfra*-R | GTGGAACTACTGGAACCTGTCTCG |
| *Pcna*-F | TTGCACGTATATGCCGAGACC |
| *Pcna*-R | GGTGAACAGGCTCATTCATCTCT |
| *Fn*-F | GGTGACACTTATGAGCGCCCTA |
| *Fn*-R | AACATGTAGCCACCAGTCTCAT |
| *CC10*-F | ATGAAGATCGCCATCACAATCAC |
| *CC10*-R | GGATGCCACATAACCAGACTCT |
| *Pten*-F | AATTCCCAGTCAGAGGCGCTATGT |
| *Pten*-R | GATTGCAAGTTCCGCCACTGAACA |
| *Hes1*-F | CAGCCAGTGTCAACACGACAC |
| *Hes1*-R | TCGTTCATGCACTCGCTG |
| *β-actin*-F | TGTCTGGCGGCACCACCATG |
| *β-actin*-R | AGGATGGAGCCGCCGATCCA |
| *Sox2*-F | CTACGCGCACATGAACGG |
| *Sox2*-R | CGAGCTGGTCATGGAGTTGT |
| *Sox9*-F | AACATGGAGGACGATTGGAG |
| *Sox9*-R | TCCCCTCAAAATGGTAATGAG |
| *Ssea1*-F | TAAGGCGCTACCAGTGTTCG |
| *Ssea1*-R | GGACGAGAACCTACCAGGG |
| *Spc-*F | CCACTGGCATCGTTGTGTAT |
| *Spc-*R | GCCATCTTCATGATGTAGCAGT |
| *T1α-*F | GCCAACTTGTAACTGCACTTGAT |
| *T1α-*R | GAAGGCACAGTGGATGCTTAG |
| *α-SM-*F | CGCTGCTCCAGCTATGTGTGA |
| *α-SM-*R | TTTGGCCCATTCCAACCATTAC |
| *β-tubulin-F* | TTCACTCTGGCTCCCGTAAG |
| *β-tubulin-R* | GTGGATTTTAGGAGGGGCT |
| *Pgp9.5-F* | GATGCTGAACAAAGTGTTGGC |
| *Pgp9.5-R* | GGAGTTTCCGATGGTCTGCTT |
| *Bmp4-F* | TTCCTGGTAACCGAATGCTGA |
| *Bmp4-R* | CCTGAATCTCGGCGACTTTTT |
| *Wnt2-F* | CTCGGTGGAATCTGGCTCTG |
| *Wnt2-R* | CACATTGTCACACATCACCCT |
| *Wnt2b-F* | CCGACGTGTCCCCATCTTC |
| *Wnt2b-R* | GCCCCTATGTACCACCAGGA |
| *Spry-F* | TCCAAGAGATGCCCTTACCCA |
| *Spry-R* | GCAGACCGTGGAGTCTTTCA |
| *Gli1-F* | CCAAGCCAACTTTATGTCAGGG |
| *Gli1-R* | AGCCCGCTTCTTTGTTAATTTGA |

**Supplemental Table 2.** **Primers list.**

The primers used in this study were listed.
